# Impact of TET2, SRSF2, ASXL1 and SETBP1 mutations on survival of patients with chronic myelomonocytic leukemia

**DOI:** 10.1186/s40164-015-0009-y

**Published:** 2015-05-20

**Authors:** Yajuan Cui, Hongyan Tong, Xin Du, Bing Li, Robert Peter Gale, Tiejun Qin, Jinqin Liu, Zefeng Xu, Yue Zhang, Gang Huang, Jie Jin, Liwei Fang, Hongli Zhang, Lijuan Pan, Naibo Hu, Shiqiang Qu, Zhijian Xiao

**Affiliations:** MDS and MPN Center, Institute of Hematology and Blood Diseases Hospital, Chinese Academy of Medical Sciences & Peking Union Medical College, 288 Nanjing Road, Tianjin, 300020 China; State Key Laboratory of Experimental Hematology, Institute of Hematology and Blood Diseases Hospital, Chinese Academy of Medical Sciences & Peking Union Medical College, Tianjin, 300020 China; Department of Hematology, The First Affiliated Hospital, ZheJiang University College of Medicine, Zhejiang, China; Department of Hematology, Guangdong General Hospital, Guangzhou, China; Hematology Research Center, Division of Experimental Medicine, Department of Medicine, Imperial College London, London, UK; Divisions of Experimental Hematology and Cancer Biology, Cincinnati Children’s Hospital Medical Center, Cincinnati, OH USA

**Keywords:** Chronic myelomonocytic leukemia, Mutation, Prognostic model

## Abstract

**Background:**

Chronic myelomonocytic leukemia (CMML) is a myeloid neoplasm classified in the myelodysplastic syndrome/myeloproliferative neoplasm (MDS/MPN) category. Molecular abnormalities are reported in about 90 % of patients with CMML. ASXL1 and SETBP1 mutations, but not TET2 or SFRS2 mutations are reported to be associated with prognosis.

**Methods:**

We studied frequency of TET2, SRSF2, ASXL1 and SETBP1 mutations in 145 patients with CMML using Sanger sequencing, and determined the prognostic factors for OS. We also identified the predictive value of ASXL1 mutations (frameshift and nonsense mutations) through comparing the Mayo Prognostic Model with the Mayo Molecular Model.

**Results:**

Forty-seven (32 %) had a mutation in TET2, 42 (29 %), a mutation in SRSF2, 65 (45 %), a mutation (nonsense and frame-shift) in ASXL1 and 26 (18 %), a mutation in SETBP1. Significant variables in multivariable analysis of survival included ASXL1 (HR = 1.99 [1.20–3.28]; *P* = 0.007), hemoglobin <100 g/L (HR = 2.42 [1.40–4.19]; *P* = 0.002) and blood immature myeloid cells (IMCs) (HR = 2.08 [1.25–3.46]; *P* = 0.005). When our patients were analyzed using the Mayo Prognostic Model median OS were not reached, 26 months and 15 months (*P* = 0.014). An analysis using the Mayo Molecular Model identified 4 cohorts with median OS of not reached, 70 months, 26 months and 11 months (*P* < 0.001). Data fitting using our patients suggest the Molecular Mayo Model has significantly higher survival predictive power compared with Mayo Prognostic Model (*P* < 0.001, −2 log-likelihood ratios of 538.070 and 552.260).

**Conclusions:**

There were high frequencies of mutations in TET2, SRSF2, ASXL1 and SETBP1 in patients with CMML. With the addition of ASXL1 frameshift and nonsense mutations, the Mayo Molecular Model fitted better than Mayo Prognostic Model of our patients.

## Introduction

Chronic myelomonocytic leukemia (CMML) is in the overlap category of myelodysplastic syndrome/myeloproliferative neoplasms (MDS/MPN) in the World Health Organization (WHO) classification. CMML is uncommon with an estimated incidence of 0.4/100,000 patients/year. In the WHO classification it is defined as persistent blood monocytes >1 × 10^9^/L, no BCR-ABL1 or PDGFRA/B mutation, <20 % myeloblasts or promonocytes in the blood or bone marrow and dysplasia in one or more myeloid lineages [1].

Molecular abnormalities are detected in about 90 % of patients with CMML [2, 3] including TET2 in 50–60 %, SRFS2 in 40–50 %, ASXL1 in 40–50 % and SETBP1 in 5–10 % [2, 4]. ASXL1 and SETBP1 mutations, but not TET2 or SFRS2 mutations are reported to be associated with prognosis [4–7].

Several models are used to predict survival of patients with CMML including: (1) the MD Anderson prognostic scoring system (MDAPS) in 213 patients [8]; (2) the CMML-specific prognostic scoring system (CPSS) in a large series of 558 patients [9]; (3) the Mayo Prognostic Model (MPM) in 226 patients [10]; (4) Groupe Francais des Myelodysplasies (GFM) in 312 patients [4]; (5) the Mayo Molecular Model (MMM) [11], and etc. A study from the Mayo Clinic reported no significant association between ASXL1 mutations (missense, nonsense and frameshift) and leukemia free survival (LFS) or overall survival (OS) [10]. In contrast a study of GFM reported a significant association between ASXL1 mutations (nonsense and frameshift) and OS [4]. A second report from the Mayo Clinic and cooperators in 466 patients using the MMM reported a significant association between ASXL1 mutation (nonsense, frameshift) and LFS and OS [11]. We studied the frequency of TET2, SRSF2, ASXL1 and SETBP1 mutations in 145 patients with CMML and compared their outcomes with those predicted in the two Mayo Clinic prognostic models.

## Results

### Patients

Baseline variable are listed in Table 1. Median age was 63 years (range, 18–85 years) and 98 (68 %) were male. Applying the WHO classification 84 (58 %) of patients were identified as CMML-1 and 61 (42 %), as CMML-2. Applying the FAB classification 51 patients (35 %) patients were classified as having CMML-MD and 94 (65 %), CMML-MP. Median WBC was 21.88 × 10^9^/L (range, 3.01–117.57 × 10^9^/L). Median platelets were 78 × 10^9^/L (4–1001 × 10^9^/L). Median hemoglobin concentration was 88.0 g/L (43.0–166.0 g/L). Three patients received a transplant, 13, decitabine and the remainder hydroxyurea and supportive treatment.

**Table 1:** Clinical and laboratory features in 145 patients with chronic myelomonocytic leukemia

Age in years, median (range)	63(18–85)
Males; n (%)	98(68)
Hemoglobin g/L, median (range)	88.0(43.0–166.0)
WBC (10^9^/L), median (range)	21.88(3.01–117.57)
ANC (10^9^/L), median (range)	7.07(0.30–66.91)
AMC (10^9^/L), median (range)	3.58(1.02–57.72)
Platelets (10^9^/L), median (range)	78(4–1001)
FAB subtypes, n (%)	
Myelodysplastic, MD	51(35)
Myeloproliferative, MP	94(65)
WHO subtypes, n (%)	
CMML-1	84(58)
CMML-2	61(42)
Mutational status	
ASXL1, n (%)	65(45)
SETBP1, n (%)	26(18)
TET2, n (%)	47(32)
SRSF2, n (%)	42(29)
^a^Acute leukemic transformation; n (%)	18(14)
^a^Deaths; n (%)	71(56)

^a^Data of acute leukemic transformation and deaths were limited to 127 patients because of 15 cases of limited updating and 3 cases of transplantation

### Spectrum and correlation of gene mutations

**Fig. 1:**
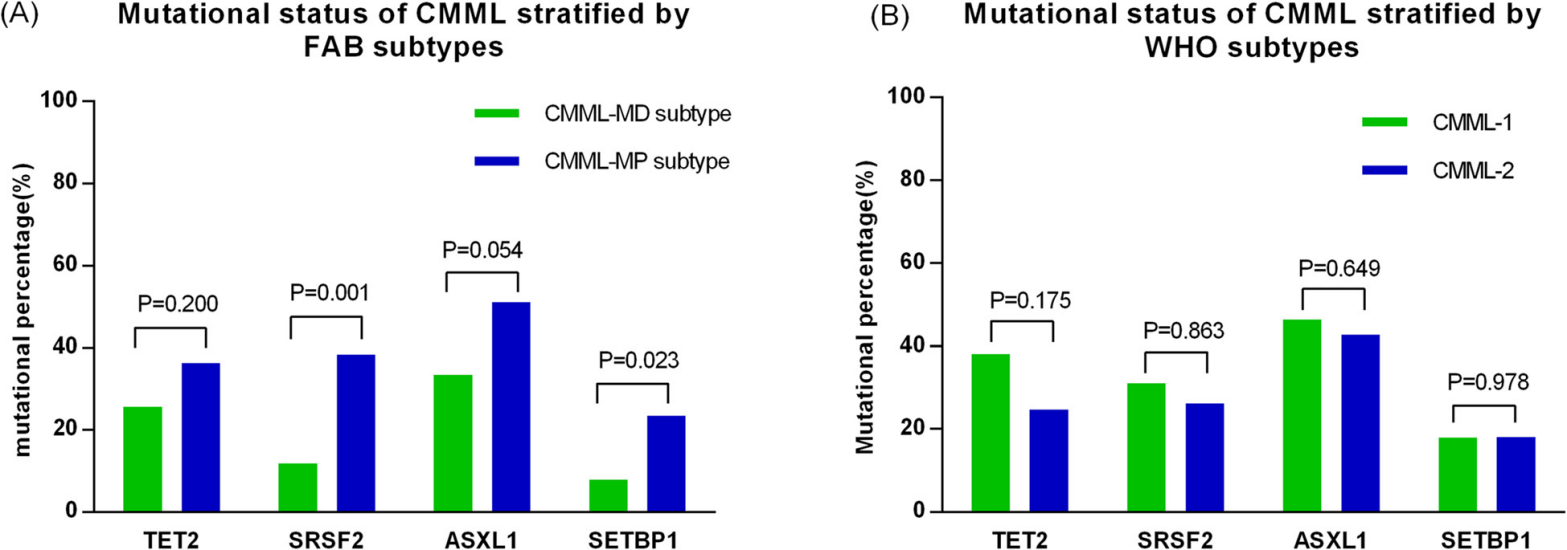
Mutational spectrum of TET2, SRSF2, ASXL1 and SETBP1 stratified by FAB and WHO subtypes. (**a**) Mutations in SRSF2 and SETBP1 were more frequent in CMML-MP than CMML-MD. (**b**) There were no significant differences in mutation frequencies of these four genes between patients with CMML-1 *vs.* those with CMML-2

**Fig. 2:**
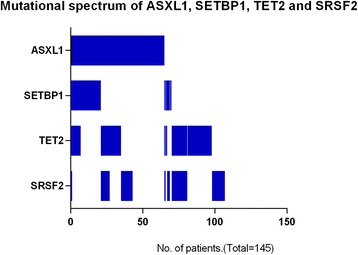
Combinations of mutations state of TET2, SRSF2, ASXL1 and SETBP1 according ASXL1 mutation in 145 patients with CMML

TET2 mutations were detected in 47 (32 %) patients, of them, 22 were frameshift mutations, 6 nonsense mutations and 19 missense mutations. SRSF2 mutations were detected in 42 (29 %) patients including 39 missense and 3 frameshift mutations. 74 patients (51 %) had an ASXL1 mutation including 59 with frameshift mutations (31 with c.1934dupG; p.G646WfsX12), 6 a nonsense mutation and 9 missense and synonymous mutations (the following analysis of ASXL1 mutations only include the frameshift and nonsense mutations due to the prognostic value of only frameshift and nonsense mutations). Missense mutations focused on a hotspot area from D868 to I871 in SETBP1 were detected 26 (18 %) patients. No mutation in these 4 genes was detected in 38 patients (26 %). Mutations in SRSF2 were more frequent in CMML-MP than CMML-MD (38 % *vs.* 12 %; *P* = 0.001). Similarly, SETBP1 mutations were more common in patients with CMML-MP compared with patients with CMML-MD (23 % *vs*. 8 %, *P* = 0.023, respectively) (Fig. 1a). There were no significant differences in mutation frequencies of these four genes between patients with CMML-1 *vs.* those with CMML-2 (Fig. 1b). Combinations of mutations according ASXL1 mutational state are shown in Fig. 2. TET2 and SRSF2 mutations were frequently concordant (*P* = 0.035). Similarly, ASXL1 and SETBP1 mutations were frequently concordant (*P* < 0.001; Table 2).

**Table 2:** Association of gene mutations

P value	SETBP1	ASXL1	SRSF2	TET2
TET2	0.113	0.147	0.035	×
SRSF2	0.823	0.949	×	
ASXL1	0.000	×		
SETBP1	×			

*P* < 0.05, mutations concomitant

*P* > 0.05, not significant

### Associations between baseline variables and mutations

Associations between baseline variables and mutations are summarized in Table 3. TET2 mutation was associated with older age (*P* = 0.005) and a greater proportion of patients with <10 % bone marrow blasts (*P* = 0.008). SRSF2 mutations was also associated with older age (*P* = 0.000), higher WBC levels (*P* = 0.027), higher absolute neutrophil levels (*P* = 0.008), higher blood monocyte levels (*P* = 0.004) and higher hemoglobin concentration (*P* < 0.001). There was no significant difference in baseline variables in patients with and without ASXL1 or SETBP1 mutations.

**Table 3:** Gene mutations and clinical characteristics

	wt vs	mut	P value
TET2			
Age(>65y), %	36.2	61.2	0.005
BM blasts(>10 %), %	37.8	17	0.008
WBC(10^9^/L), median, range	12.96(3.05–98.03)	16.00(3.01–70.50)	0.961
ANC(10^9^/L), median, range	5.83(0.30–66.91)	8.78(1.12–28.64)	0.617
AMC(10^9^/L), median, range	3.06(1.02–18.00)	3.13(1.06–17.63)	0.874
Hb(g/L), median, range	91.0(43.0–158.0)	88.0(43.0–166.0)	0.409
PLT(10^9^/L), median, range	68.0(3.8–895.0)	87.0(9.0–1001.0)	0.866
SRSF2			
Age(>65y), %	24.2	64.1	0.000
BM blasts(>10 %), %	33	26.2	0.361
WBC(10^9^/L), median, range	11.47(3.01–98.03)	21.35(5.06–70.50)	0.007
ANC(10^9^/L), median, range	4.92(0.30–66.91)	13.55(1.75–33.90)	0.008
AMC(10^9^/L), median, range	2.29(1.02–18.00)	3.72(1.16–17.63)	0.004
Hb(g/L), median, range	84.0(43.0–166.0)	106.5(56.0–166.0)	0.000
PLT(10^9^/L), median, range	67.5(3.8–895.0)	100.0(9.0–1001.0)	0.392
ASXL1			
Age(>65y), %	52.3	53.8	0.863
BM blasts(>10 %), %	28.8	38.8	0.393
WBC(10^9^/L), median, range	11.47(3.01–92.40)	20.10(3.62–98.03))	0.123
ANC(10^9^/L), median, range	5.17(0.30–66.91)	10.45(0.46–56.66)	0.052
AMC(10^9^/L), median, range	2.52(1.06–18.00)	3.20(1.02–14.82)	0.082
Hb(g/L), median, range	92.0(44.0–166.0)	87.5(43.0–158.0)	0.187
PLT(10^9^/L), median, range	69.0(6.6–1001.0)	90.5(3.8–633.0)	0.487
SETBP1			
Age(>65y), %	57.7	52.1	0.605
BM blasts(>10 %), %	29.4	38.5	0.415
WBC (10^9^/L), median, range	12.69(3.01–98.03)	20.46(6.60–49.10)	0.173
ANC (10^9^/L), median, range	5.50(0.30–66.91)	11.75(2.56–33.90)	0.079
AMC (10^9^/L), median, range	2.62(1.06–18.00)	3.25(1.02–11.03)	0.413
Hb(g/L), median, range	88.5(43.0–166.0)	90.5(52.0–137.0)	0.622
PLT(10^9^/L), median, range	74.25(5.0–1001.0)	118.5(3.8–534.0)	0.337

### Prognostic impact of mutations

Follow-up data were available for 127 patients (88 %) with a median follow-up interval of 13 months (range, 1–95 months), 18 patients (14 %) transformed to acute leukemia and 71 (56 %) died (including 15 cases died after acute leukemia transformation).

**Fig. 3:**
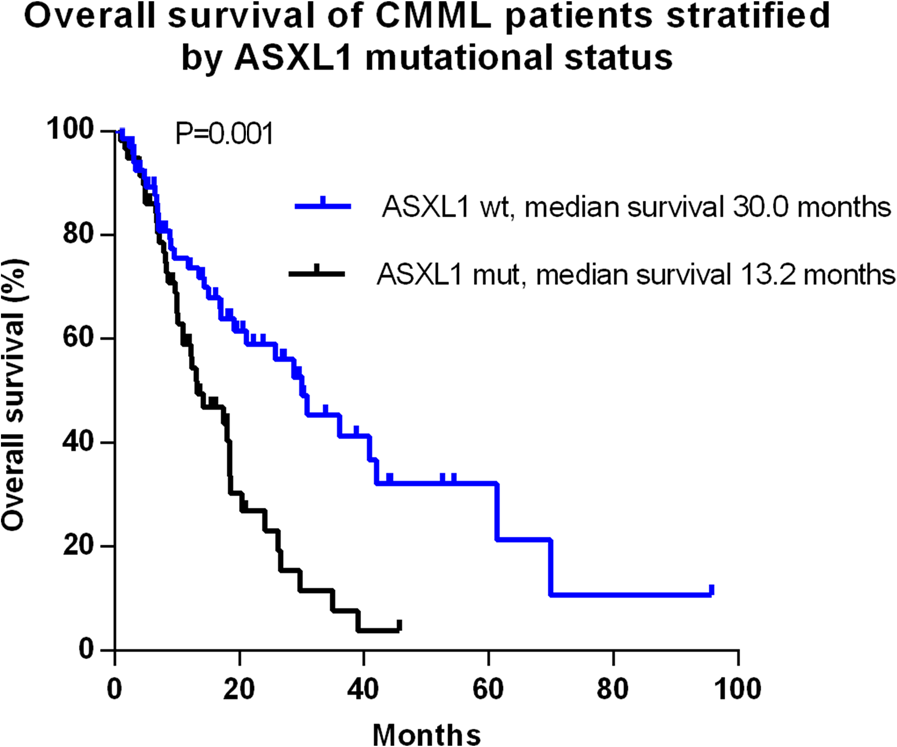
Survival data for 127 patients with CMML stratified by ASXL1 mutational status (frameshift and nonsense mutations only)

Variables significantly associated with survival in multivariable analysis included ASXL1 (HR = 1.99 [1.20–3.28]; *P* = 0.007) (Fig. 3), hemoglobin <100 g/L (HR = 2.42 [1.40–4.19]; *P* = 0.002) and blood immature myeloid cells (IMCs) (HR = 2.08 [1.25–3.46]; *P* = 0.005) (Table 4).

**Table 4:** Multivariable overall survival analysis for 127 patients with CMML

Parameters	HR	95%CI	P value
Hb < 100 g/L vs ≥100 g/L	2.42	1.40–4.19	0.002
IMCs presence vs absence	2.08	1.25–3.46	0.005
ASXL1 mut vs wt	1.99	1.20–3.28	0.007

**Fig. 4:**
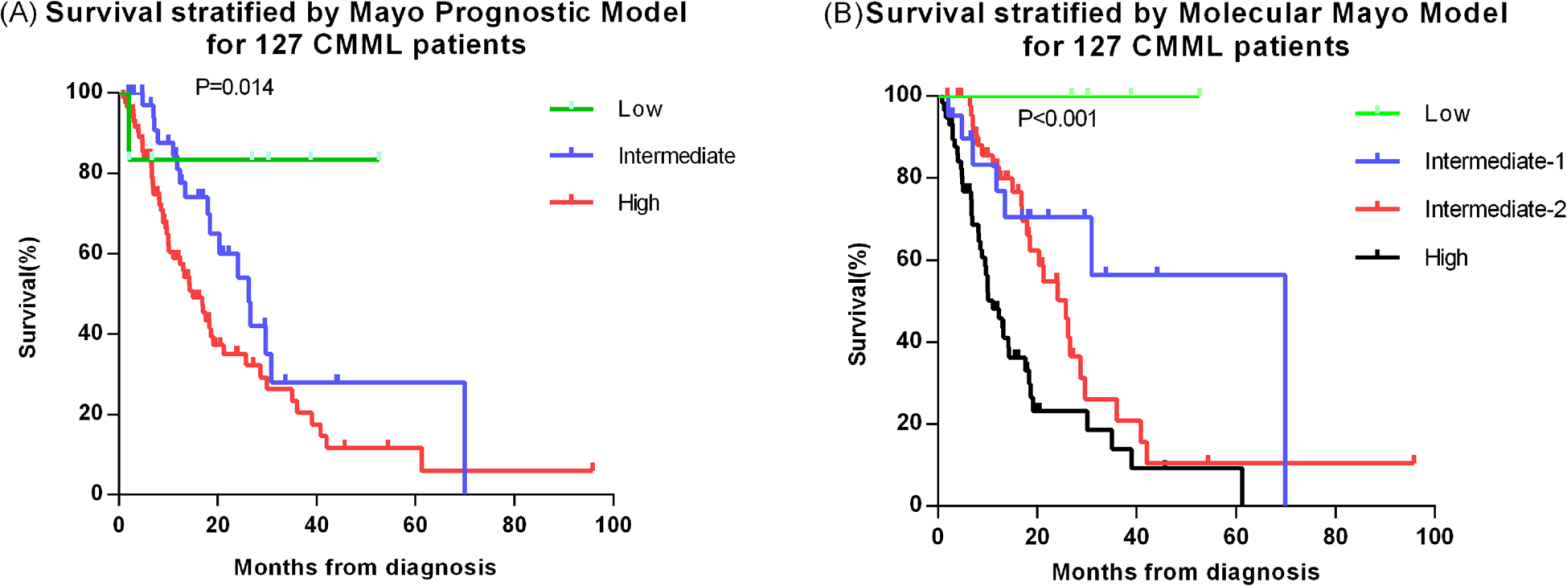
Survival data for 127 patients with CMML stratified by the Mayo prognostic model and the Molecular Mayo model. (**a**) Median survivals using the Mayo Prognostic Model were not reached, 26 months, and 15 months. (**b**) Median survivals using the Molecular Mayo Model were not reached, 70 months, 26 months and 11 months

To clarify the prognostic impact of ASXL1 mutations on survival we evaluated survival of subgroups based on the Mayo Prognostic Model and Molecular Mayo Model. Median survivals using the Mayo Prognostic Model were not reached, 26 months (95 % CI, 19–34 months) and 15 months (95 % CI, 11–19 months) (*P* = 0.014) (Fig. 4a). Median survivals using the Molecular Mayo Model were not reached, 70 months (95 % CI not available), 26 months (95 % CI, 20–32 months) and 11 months (95 % CI, 7–15 months) (*P* < 0.001) (Fig. 4b). Data fitting using our patients suggest the Molecular Mayo Model has significantly higher survival predictive power compared with Mayo Prognostic Model (*P* < 0.001, −2 log-likelihood ratios of 538.070 and 552.260).

## Discussion

Mutations in patients with CMML typically involve epigenetic regulator genes, spliceosome component pathway genes, genes controlling transcription factors and signaling regulator genes [12, 13]. Itzykson R et al. [3] have recently demonstrated that patients with increased variant allelic frequency of TET2 are more likely to demonstrate granulomonocytic hematopoietic skewing on the basic of early clonal dominance. The diverse combinations of mutations detected in CMML suggest multi-step pathogenesis of the disease in some cases. For example, although TET2 and ASXL1 mutations may be independent drivers of CMML in some patients [3], combined mutations of TET2 and SRSF2 and of ASXL1 with SETBP1 are consistent with a two-step ‘linear’ model of CMML development [14]. The complex and diverse mutation spectrum detected by us and others in patients with CMML suggest complexity from driver mutation to clonal evolution to clonal dominance and finally to the disease. This complex pattern may account for the considerable clinical diversity of CMML.

Recurrent mutations of SETBP1 gene are detected in about 80 % of patients with chronic neutrophilic leukemia (CNL) [15] and in about 25 % of patients with atypical chronic myeloid leukemia [16]. Mutations in SETBP1 decrease PP2A activity resulting in increased proliferation [16, 17]. This increase could explain why patients with SETBP1 mutations in our study were more likely to be in the CMML-MP subtype *vs.* the CMML-MD subtype.

Patients with SRSF2 mutation were also more likely to be in the CMML-MP. SRSF2 mutation was also associated with increased age and a higher hemoglobin concentration as reported previously [7]. Interestingly, Yoshida K et al. [18] reported splice-gene mutations introduced into normal hematopoietic cells cause a proliferation defect *in vitro* and a competitive disadvantage *in vivo.*

ASXL1 regulates epigenetic functions (histone and chromatin modification) and transcription. ASXL1 mutations are detected in patients with myelodysplastic syndrome (MDS), primary myelofibrosis, CMML and acute myeloid leukemia. Most ASXL1 mutations are frameshift mutations [4, 10]. We also found a predominance of frameshift mutations (31 of c.1934dupG; p.G646WfsX12), 59 *vs.* only 6 nonsense mutations. There is controversy whether c.1934dupG; p.G646WfsX12 is real or is a PCR artifact [19]. However, recent data indicate patients with c.1934dupG; p.G646WfsX12 have a similar clinical phenotype to patients with other ASXL1 mutations [4, 20]. Based on these data we consider c.1934dupG; p.G646WfsX12 *bona fide* mutations. In Mayo Clinic study nonsense/frame-shift ASXL1 mutations were associated with worse survival whereas they were not together with missense mutations [10, 11]. We found nonsense/frame-shift ASXL1 mutations were significantly associated with survival in the final Cox model along with hemoglobin concentration <100 g/L and presence of blood IMCs.

There were several prognostic systems for CMML based either on the FAB classification of CMML or using systems designed for other diseases including a cytogenetics-based risk-stratification [21]. Other prognostic models were developed specifically for patients with CMML. For example, the MDAPS model uses data on hemoglobin concentration, blood IMCs, absolute lymphocyte counts (ALC) and percent bone marrow blasts [22] to define risk categories. The G-MDAPS, developed for patients with *de novo* and secondary MDS, and CMML, uses age, performance score, platelet level, hemoglobin concentration, bone marrow blasts, cytogenetics data and RBC-transfusion state to define risk cohorts [23]. The Mayo Prognostic Model used WBC count, platelet count, hemoglobin concentration, and blood IMCs to define risk cohorts. We tested our survival data against to Mayo Prognostic Model and Mayo Molecular Model to determine the best fit. We found the Mayo Molecular Model, with the addition of ASXL1 mutations (nonsense and frameshift mutations) based on Mayo Prognostic Model, had better predictive power compared with the Mayo Prognostic Model.

## Conclusions

In summary, we found a high frequency of mutations in TET2, SRSF2, ASXL1 and SETBP1 in patients with CMML. Often there were several mutations in a person and we found some significant association between mutation spectrum and clinical and laboratory variables. We also found the Mayo Molecular Model best fitted the survival experience of our patients.

### Patients and methods

#### Patients

A hundred and forty five consecutive patients ≥16 years of age diagnosed with CMML at 3 centers in China from January, 2007 to December, 2014 were enrolled. None received prior therapy or exposed to environmental carcinogens. Baseline variables at diagnosis or referral were analyzed and patients classified into CMML-1 or CMML-2 according to the 2008 WHO criteria [1]. Myelodysplastic and myeloproliferative subtypes (CMML-MD and CMML-MP, respectively) were defined according to FAB criteria [24]. The study was approved by the Ethical Committees of the Institute of Hematology, Chinese Academy of Medical Sciences (CAMS) and Peking Union Medical College (PUMC) following principles of the Declaration of Helsinki and all patients gave written informed consent.

### PCR and Sanger sequencing

Genomic DNA was extracted using the AxyPrep blood genomic DNA Miniprep kit (Axygen Biosciences, AP-MN-BL-GDNA-250 Union City, CA, USA) from bone marrow cells. Oligonucleotide primers for TET2 (exon 3 to 17), SRSF2 (exon 1, covering amino acid Pro95), ASXL1 (exon 12) and SETBP1 (amino acid 800 to 935) were used described previously [25, 26]. All PCR products were confirmed by 1 % agarose gel, purified using QIAquick Spin Kit (Qiagen, Santa Clarita, CA, USA) and sequenced using two ABI PRISM 3730xl DNA Analyzers (Applied Biosystems, Foster City, CA, USA). Sequencing was bi-directional.

### Statistical analyses

Numerical variables are presented as medians and ranges. Categorical variables are described as counts and relative frequencies (%). Comparisons between categorical variables were performed using *χ*^2^ tests. Comparisons between continuous variables were performed using the Mann–Whitney *U*-test. Survival was analyzed by the Kaplan–Meier method and compared using the log-rank test. A Cox model was used to identify the prognostic variables. The above analyses were conducted with SPSS version 17.0. The likelihood ratio test was used to compare the Mayo Prognostic Model [10] and MMM [11] conducted with SAS 9.3. All P-values are two-tailed, and statistical significance was set at *P* < 0.05.

## Abbreviations

CMML: Chronic myelomonocytic leukemia
MDS/MPN: Myelodysplastic syndrome/ myeloproliferative neoplasms
MDAPS: MD Anderson prognostic scoring system
CPSS: CMML-specific prognostic scoring system
MPM: Mayo prognostic mode
GFM model: Groupe Francais des myelodysplasies model
MMM: Mayo molecular model
LFS: Leukemia free survival
OS: Overall survival
WBC: White blood cell count
ANC: Absolute neutrophil count
AMC: Absolute monocyte count
SRSF2: Serine/arginine-rich splicing factor 2. ASXL1, additional sex combs 1 gene
SETBP1: SET binding protein 1
Hb: Hemoglobin
PLT: Platelet count
IMCs: Blood immature myeloid cells
